# Editorial: Food Additives, Cooking and Processing: Impact on the Microbiome

**DOI:** 10.3389/fnut.2021.731040

**Published:** 2021-08-02

**Authors:** Xi Yu, Tao Zuo

**Affiliations:** ^1^Faculty of Medicine, Macau University of Science and Technology, Macau, China; ^2^Guangdong-Hong Kong-Macau Joint Laboratory for Contaminants Exposure and Health, Guangzhou, China; ^3^Guangdong Research Institute of Gastroenterology, The Sixth Affiliated Hospital of Sun Yat-sen University, Guangzhou, China; ^4^Center for Fecal Microbiota Transplantation, The Sixth Affiliated Hospital of Sun Yat-sen University, Guangzhou, China

**Keywords:** gut microbiome, food additives, cooking, food processing, diet

“You are what you eat” has recently become a prevailing proverb describing the relationship between human beings and the foods they consume every day, along with the enormous studies centered on gut microbiome linking human health and diet. Disruption of a “healthy” microbiome can contribute to, even lead to, the occurrence and/or progression of chronic non-communicable diseases as well as infectious diseases [(1, 2); Zhou et al.]. There is increasing evidence that a person's microbiome is largely impacted by the foods he/she eats among other environmental factors (3–5). In addition, not only the food itself, but also how we eat and how the food is cooked/processed, can affect our gut microbiome substantially. Different food cooking and processing habits (such as: western, eastern, or endemic; barbecued, steamed or fried; rare-, medium- or well-cooked; spicy-, sour-, or sweet-flavored) can alter both the macroscopic and microscopic structures of the constituent ingredients and nutraceuticals in a meal, thus leading to a change in how foods are digested, consumed, metabolized through the gut microbiome (He et al.).

To date, people have the awareness to plan their diets as per the food pyramid in order to promote human health (6). Indeed, beyond a simplistic action to control the total calories and the balance of different food categories consumed (fruits, vegetables, meats, grains, and dairy products) in our daily diet regimen, we also need to isolate the effect of the gut microbes on human health in relation to the diet. Studies have shown that the way how raw food materials are processed and use of food additives can all have a substantial impact on the gut microbiota and hence on the human health (Su and Liu). For instance, it is known that fermentation is a commonly employed food-processing approach which can breakdown large molecules in food into smaller ones and therefore change both its aroma and nutrition profile (7). In this collection, Shah et al. interestingly discovered that long term consumption of sourdough pasta, a fermented food, could lead to a change in the fungal community of one's gut microbiome, while the glycemic properties of the food, the testees' bacterial gut microbiota and their incretin responses are not affected by the fermentation process of the sourdough. The interactions between food, gut microbiome and host health are multi-lateral and multi-directional. A recent study conducted by Malik et al. revealed that the presence of various bacteria, including *Lactobacillus rhamnosus* and *Escherichia coli*, can remediate the detriment on the gut epithelium caused by food additives. Considering the increasingly diversified food-processing techniques and ever-evolving industry of novel class of food additives, it warrants continuous investigation into the effects of food-processing in human health in association with the gut microbiome.

With the assistance of massive experimental investigations, the impacts of cooking, food processing, and food additives on microbiome are being unveiled in recent years. Moreover, understanding how food affects microbial colonization and composition across different stages of life is also important (Dong et al.; Chen et al.). Building on these knowledge, adventurous explorations are being made by scientists and clinicians in the fields of food, microbiology, and medicine, aiming to improve the gut inter-connecting food, microbiome, and human health. Among them, rational use and combination of food components, probiotics and prebiotics, fecal microbiota transplantation, and precision microbial supplementation are trending (8, 9). However, as one critical piece of the food-health puzzle, the long-term effects of processed/cooked foods, food components, and food preservatives/additives in human health and gut microbiome as well as the mechanisms behind that are yet to be further unveiled. Overall, to maintain a healthy gut microbiome, one needs to choose appropriate food combinations and cooking/processing habits.

## Author Contributions

XY: investigation, writing-original draft preparation, project administration, and funding acquisition. TZ: resources and writing-review and editing. All authors contributed to the article and approved the submitted version.

## Conflict of Interest

The authors declare that the research was conducted in the absence of any commercial or financial relationships that could be construed as a potential conflict of interest.

## Publisher's Note

All claims expressed in this article are solely those of the authors and do not necessarily represent those of their affiliated organizations, or those of the publisher, the editors and the reviewers. Any product that may be evaluated in this article, or claim that may be made by its manufacturer, is not guaranteed or endorsed by the publisher.
